# Supplementary figures and images for: ReptiLearn: An automated home cage system for behavioral experiments in reptiles without human intervention
Source: PLoS Biol. 2024 Feb 29;22(2):e3002411. doi: 10.1371/journal.pbio.3002411 (PMC10931465; doi:10.1371/journal.pbio.3002411)

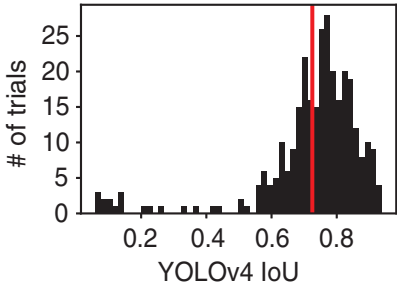

Supplement: S1 Fig — YOLOv4 intersection-over-union (IoU) distribution over a validation set consisting of 400 images sampled uniformly from video data of 4 animals and indicating good overlap with animal head. Individual numerical values are provided in S1 Data. (PDF) [file pbio.3002411.s001.pdf]

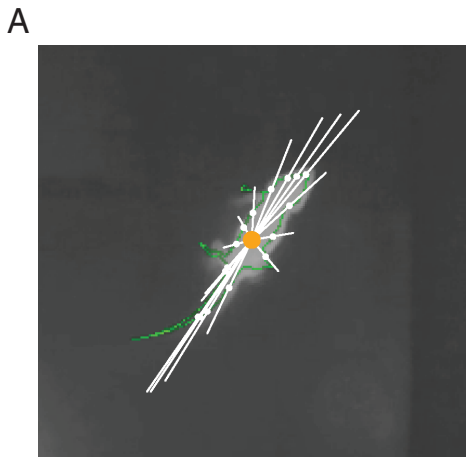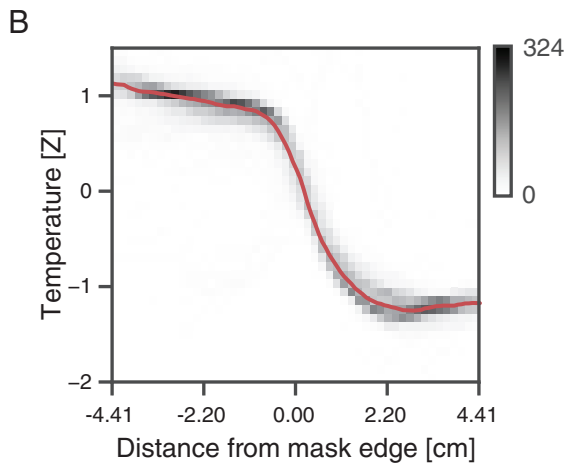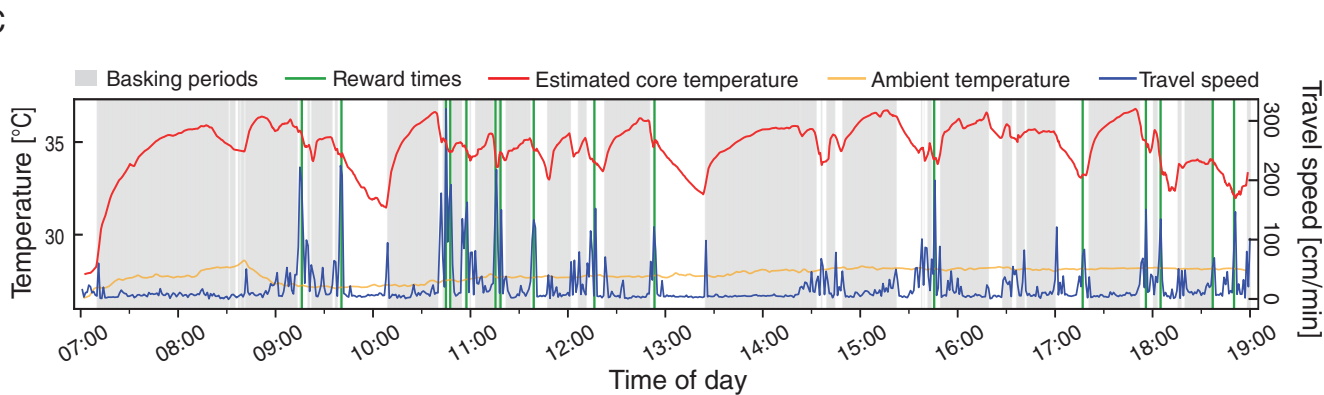

Supplement: S2 Fig — (A) Construction of line segments. An image of a lizard taken from the thermal camera is shown at the background. Line segments (white) were extended from the animal mask’s center of mass (orange) outwards to the direction of each animal edge point (green). The length of each line segment was twice the distance from the center of mass and each edge point (white dots). (B) Density plot of the temperature at each distance along the line segments. The red line shows the median temperature gradient across all frames. A sample of uniformly selected 346 thermal video frames measured for 1 day is analyzed. (C) Movement dynamics (travel speed, blue) and corresponding estimated core temperatures (red, with ambient temperature in orange) as well as reward times (green) and basking periods (gray) measured over a single day (taken from Fig 3D). Individual numerical values are provided in S1 Data. (PDF) [file pbio.3002411.s002.pdf]

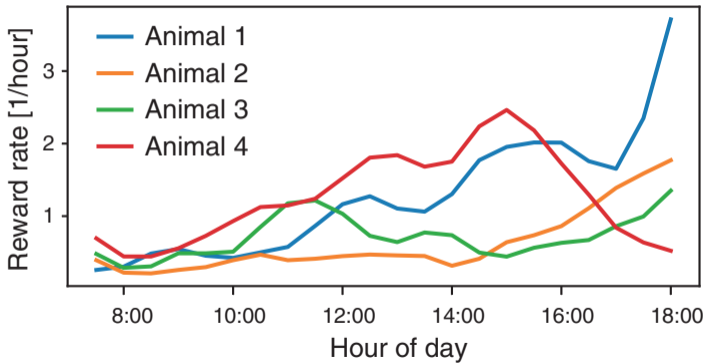

Supplement: S3 Fig — Reward times were collected (as in Fig 4C), convoluted with a normalized Gaussian (std = 30 min) and averaged over all experiment days. Individual numerical values are provided in S1 Data. (PDF) [file pbio.3002411.s003.pdf]

A

Area 1

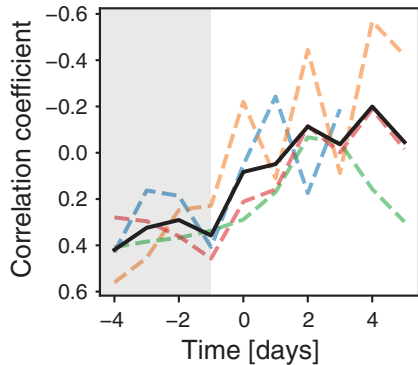

B

Area 2

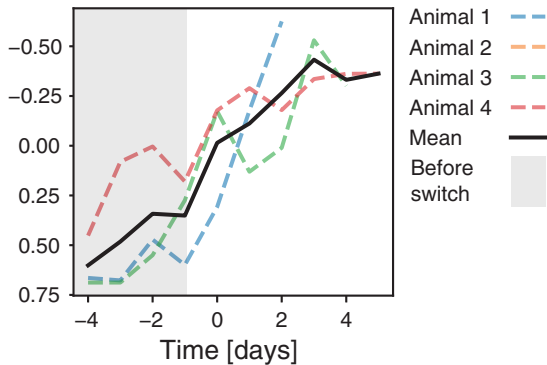

Supplement: S4 Fig — (A) Correlation coefficient of ΔER decay as a function of distance from reinforced area 1 (as in Fig 4G), calculated for each day separately (day zero marks the first day of area 2 reinforcement, gray shade marks days before reinforcement of area 1). Black line marks the average over animals. (B) Same as (A) but for reinforced area 2. Animal 2 did not complete the reversal to area 2 and was excluded from this analysis. Individual numerical values are provided in S1 Data. (PDF) [file pbio.3002411.s004.pdf]

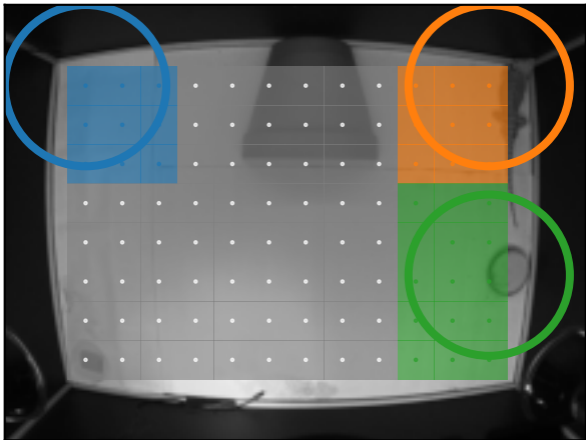

Supplement: S5 Fig — Each dot represents the center of an area with the same shape and size as the reinforced areas. Green, orange, and blue circles show the area of the feeder, the second and the third reinforced areas, respectively. Colored rectangles represent the areas neighboring each of the reinforced areas (marked in Fig 4G–4I). (PDF) [file pbio.3002411.s005.pdf]

A

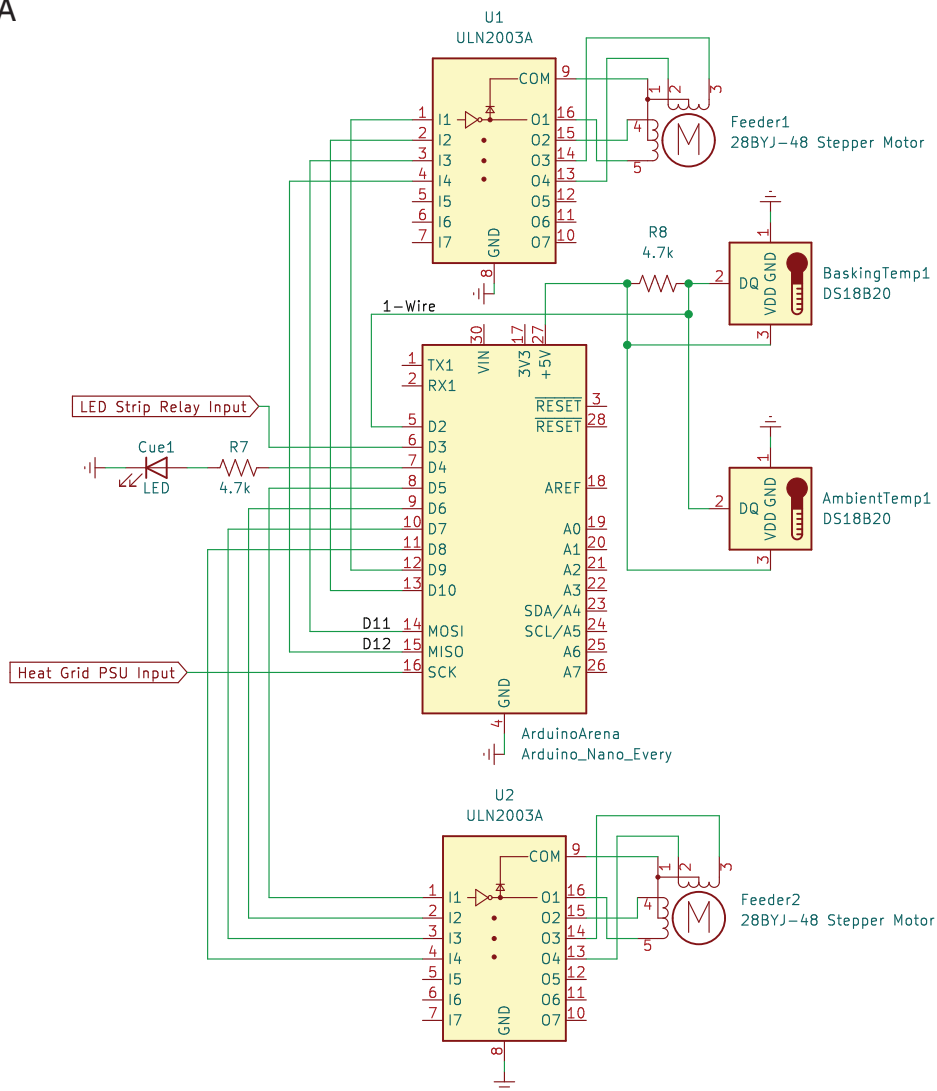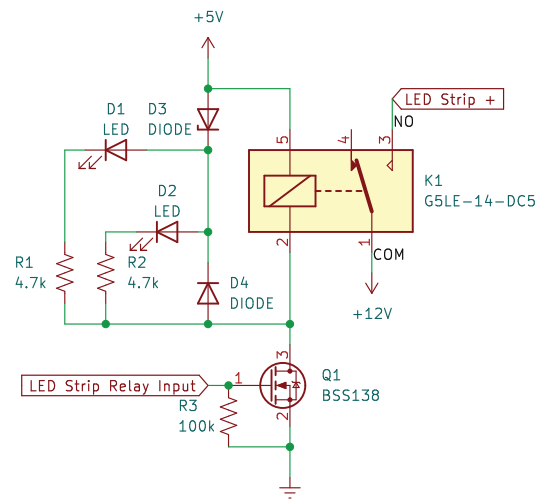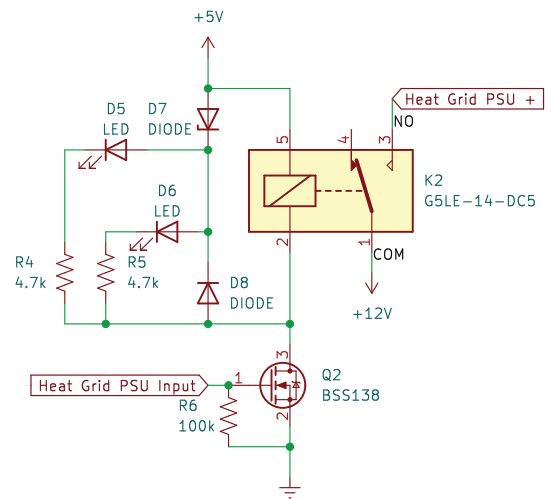

B

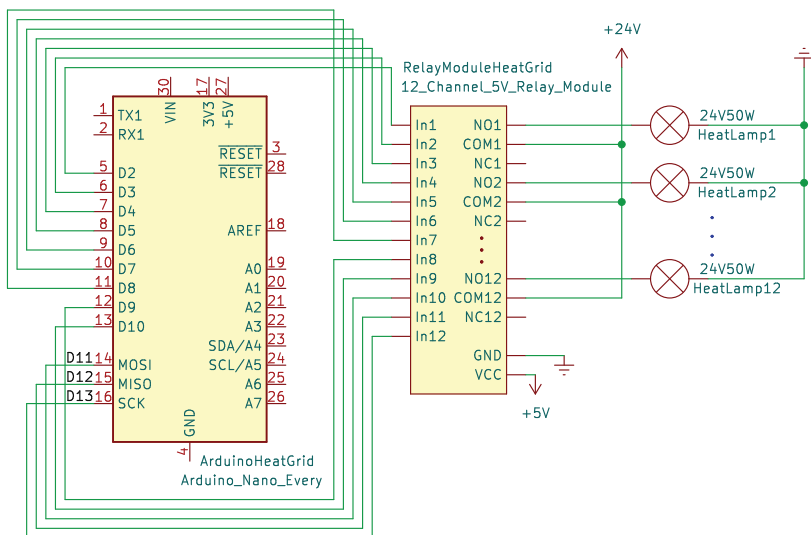

C

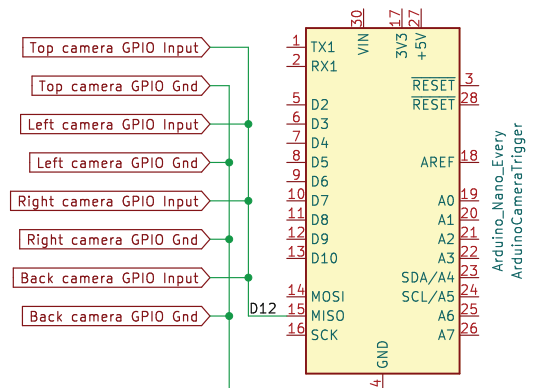

Supplement: S6 Fig — (A) An Arduino board connected to 2 feeders, 2 temperature sensors, an LED strip relay module, a cue LED, and a relay module controlling the heat grid’s power supply unit. (B) A second Arduino board connected to a 16-channel relay module that controls each of the 12 heat lamps individually. (C) A third Arduino board was responsible for synchronizing image acquisition by sending TTL pulses to GPIO inputs of 4 cameras in parallel. (PDF) [file pbio.3002411.s006.pdf]

A

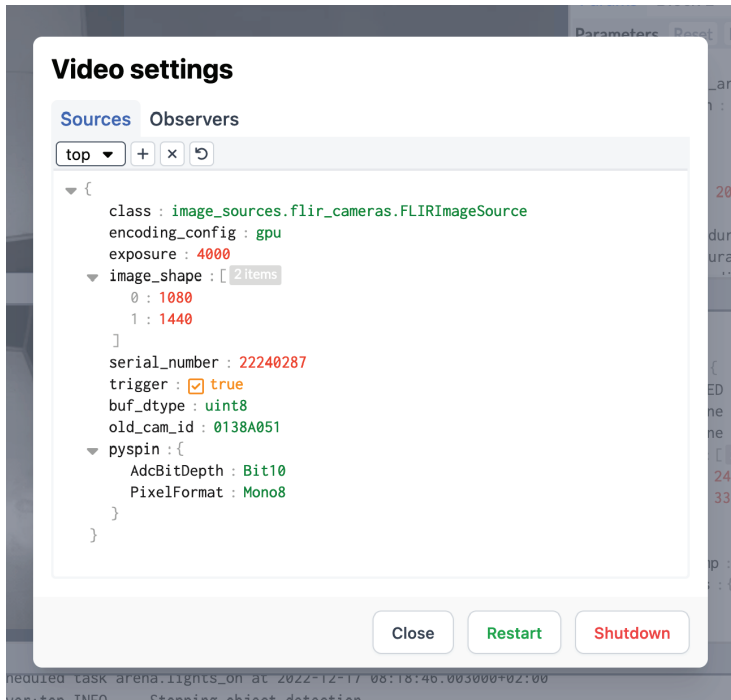

B

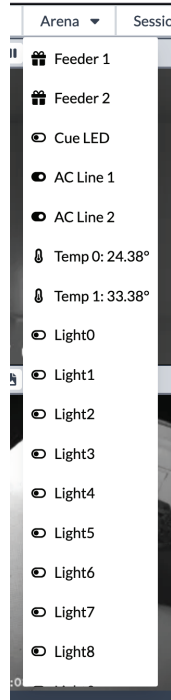

C

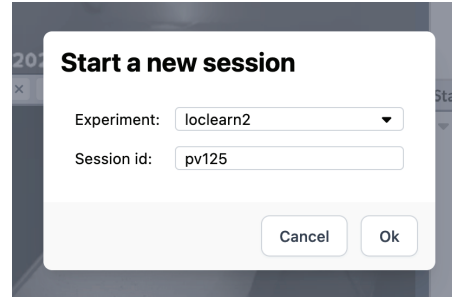

D

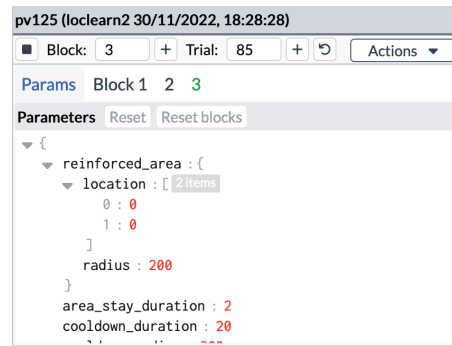

Supplement: S8 Fig — (A) Video settings window showing the parameters of an ImageSource. (B) New session dialog. The session uses the spatial learning Experiment class found in module/system/experiments/loclearn2.py. Session id determines the directory name in which data is to be stored. (C) The arena menu listing every configured arena controller interface. Feeder items can be clicked on to release a reward. Toggle interface items can be switched on or off. Sensor interface items display their most current measurement. (D) Session UI section displaying the current session name and the time of creation at the top. Located below is the session control bar that allows to start and stop the experiment and to control the current trial and block. Session and block parameters can be set using the editors in the bottom tabs. (PDF) [file pbio.3002411.s008.pdf]

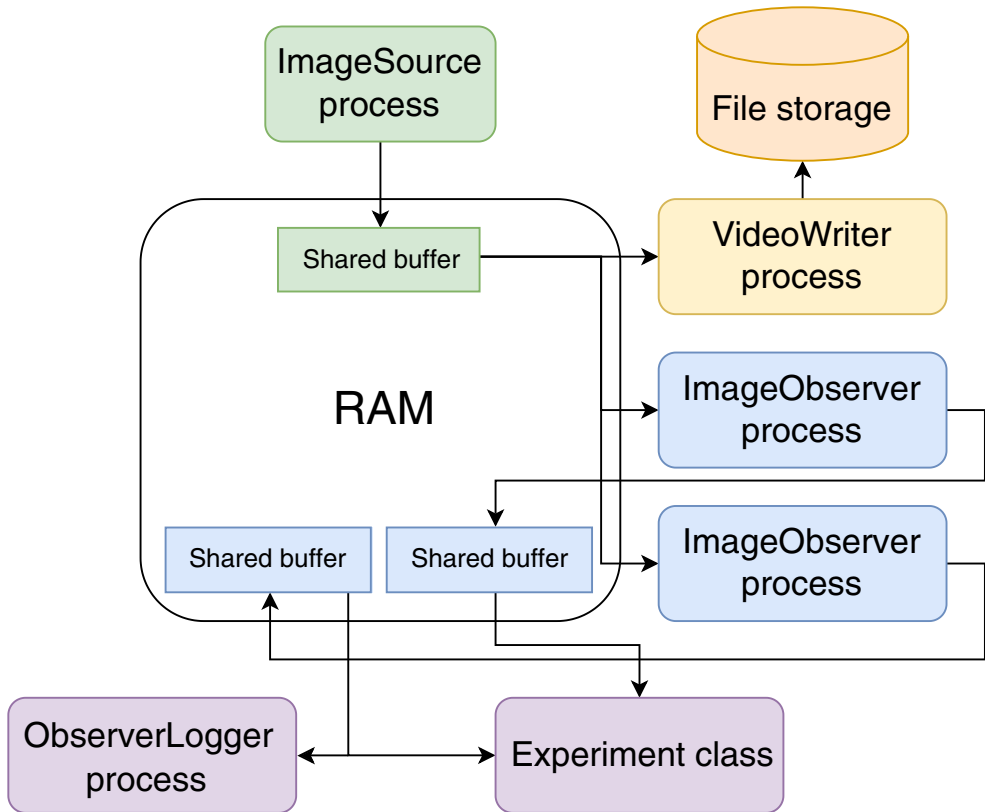

Supplement: S9 Fig — ImageSource objects acquire images and store them in shared memory buffers (green) together with timestamps. Each ImageObserver object is tied to an ImageSource and is notified when new data is written to the shared buffer (green). It processes the data and outputs a result to another shared buffer (blue). The Experiment class can then access these data through a simple API. ObserverLogger objects can access ImageObserver buffers directly and log any new results to a file or database. VideoWriter objects are specialized ImageObservers that encode and write ImageSource buffer data to video files. (PDF) [file pbio.3002411.s009.pdf]
