# Supplementary material for: ReptiLearn: An automated home cage system for behavioral experiments in reptiles without human intervention
Source: PLoS Biol. 2024 Feb 29;22(2):e3002411. doi: 10.1371/journal.pbio.3002411 (PMC10931465; doi:10.1371/journal.pbio.3002411)
Supplement: S7 Fig — The software consists of an image processing and video recording system, an Experiment class controlling the current experiment session, a state store used for synchronizing different processes, data loggers, and an MQTT client responsible for communicating with the arena controller, touch screen app, and other external software. The HTTP/WebSocket server facilitates real-time monitoring and control of the software. The arena controller handles communication with Arduino boards that control arena hardware components. (PDF) [file pbio.3002411.s007.pdf]

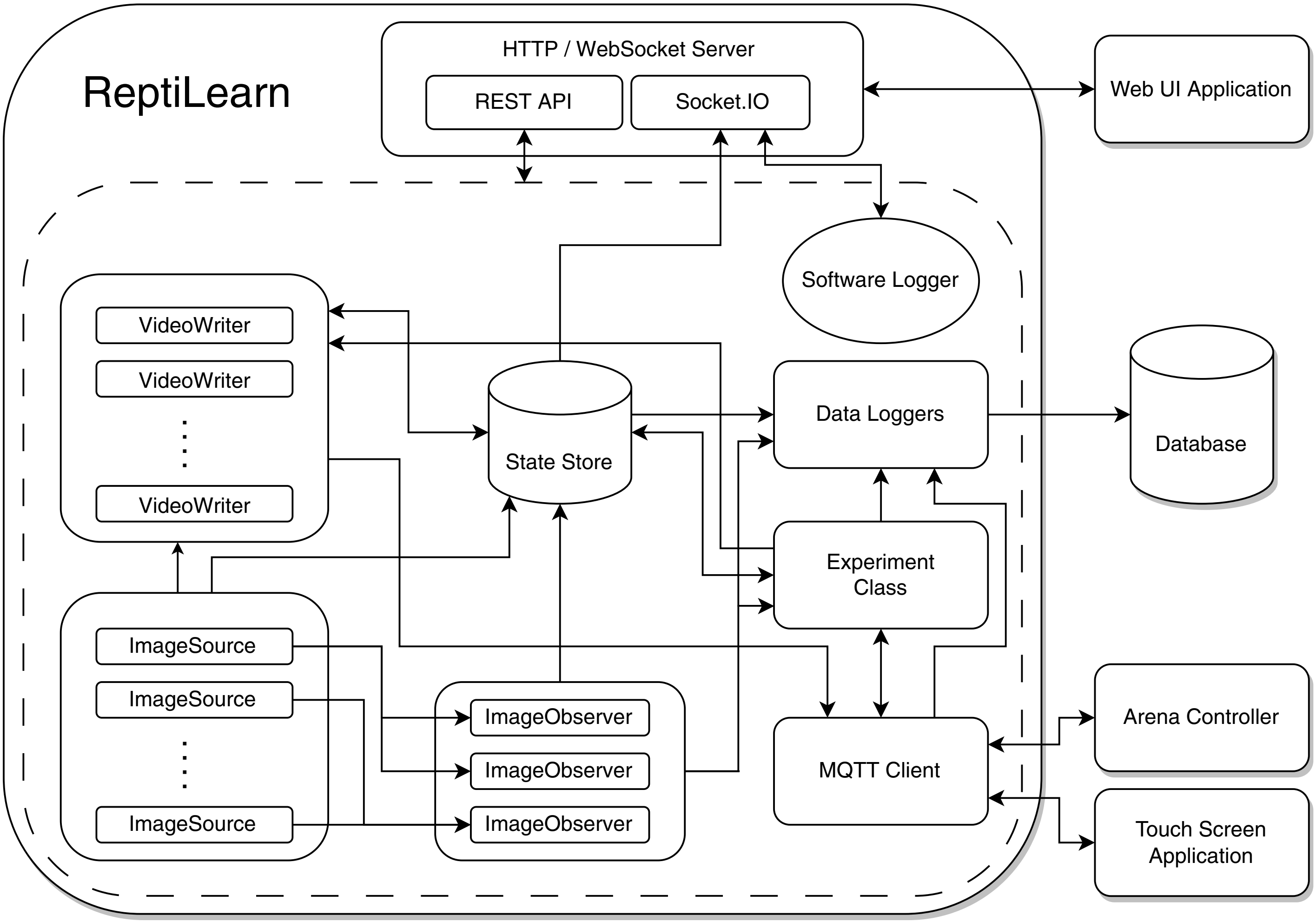
ReptiLearn
HTTP / WebSocket Server
REST API
Socket.IO
Web UI Application
Software Logger
VideoWriter
VideoWriter
VideoWriter
State Store
Data Loggers
Database
Experiment Class
ImageSource
ImageSource
ImageSource
ImageObserver
ImageObserver
ImageObserver
MQTT Client
Arena Controller
Touch Screen Application
