# Supplementary material for: ReptiLearn: An automated home cage system for behavioral experiments in reptiles without human intervention
Source: PLoS Biol. 2024 Feb 29;22(2):e3002411. doi: 10.1371/journal.pbio.3002411 (PMC10931465; doi:10.1371/journal.pbio.3002411)
Supplement: S1 Table — Mann–Whitney U statistics for the distributions of correlation coefficients of the difference in entry rate as a function of distance from each area, for all simulated and real areas. Feeder areas were excluded from the correlation calculation. (DOCX) [file pbio.3002411.s011.docx]

| **Animal** | **Area 2** | | **Area 3** |
| --- | --- | --- | --- |
|  | **Blocks 1→2** | **Blocks 2→3** | **Blocks 2→3** |
| **1** | U=781, p=1e-06  *** | U=295, p=0.23 | U=783, p=8.9e-07  *** |
| **2** | U=748, p=7.6e-06  *** | U=2, p=1e-06  *** | U=447, p=0.49 |
| **3** | U=610, p=0.0061  *** | U=6, p=1.3e-06  *** | U=644, p=0.0015  ** |
| **4** | U=679, p=0.00031  *** | U=222, p=0.034  * | U=738, p=1.4e-05  *** |

**Supplementary Table 1**. Mann-Whitney U statistics for the distributions of correlation coefficients of the difference in entry rate as a function of distance from each area, for all simulated and real areas. Feeder areas were excluded from the correlation calculation.
