# Supplementary material for: ReptiLearn: An automated home cage system for behavioral experiments in reptiles without human intervention
Source: PLoS Biol. 2024 Feb 29;22(2):e3002411. doi: 10.1371/journal.pbio.3002411 (PMC10931465; doi:10.1371/journal.pbio.3002411)
Supplement: S2 Table — All components, including the visible light cameras, are relatively low cost. An exception to this is the PC which is necessary to facilitate real-time processing. Another exception is the thermal camera that is not required if thermal monitoring is not a part of the experimental design. (DOCX) [file pbio.3002411.s012.docx]

| **Component** | **Description** | **Price Per Unit (USD)** |
| --- | --- | --- |
| Arena frame | Aluminum profile | $24 / 2 meters |
| Arena frame connectors | Nuts, bolts, washers and angle brackets | $127 |
| Arena walls & floor | 2 Alucobond sheet 122cm x 244cm gray matte | $98 |
| Side camera | FLIR Firefly S - FFY-U3-16S2M-S | $225 |
| Side camera lens | Boowon 6" - BW60BLF | $17 |
| Top camera | FLIR Blackfly - BFS-U3-16S2C-CS | $364 |
| Top camera lens | Computar A4Z2812CS-MPIR, 2.8mm-10mm 1/2.7", CS mount lens | $109 |
| Camera holder | NOGA Modular holders LC6100 | $124 |
| Arena computer | Intel Core i7-11700K CPU, 32GB DDR4 memory, NVIDIA GEFORCE RTX 3080 Ti GPU, 500GB SAMSUNG 980 M.2 NVME SSD, and a 2TB 7200 RPM HDD | $3,245 |
| Arduino Microcontrollers | Arduino Nano Every | $20 |
| Relay board (lights and heat lamps) | Pololu Basic SPDT Relay Carrier with 5VDC Relay (Assembled) - Omron G5LE-14-DC5 SPDT | $9 |
| Reward feeder | EVNICE EV200GW fish feeder | $51 |
| LED Strip | 4 meter, 12V Cold-White approx. 1A/meter | $20 |
| IR Heat Lamp | [JLT-E therapeutic apparatus infrared lamp 24V50W GZ6.35, 24V 50W reflector lamp](https://www.aliexpress.us/item/2255801026123422.html?spm=a2g0o.productlist.0.0.3b561783JmP4VQ&algo_pvid=f1c2b25b-032a-41a0-b267-9318c73811c5&algo_exp_id=f1c2b25b-032a-41a0-b267-9318c73811c5-25&pdp_ext_f=%7B%22sku_id%22%3A%2210000015319775871%22%7D&gatewayAdapt=glo2usa4itemAdapt) | $22 |
| Lamp Holder | MULTICOMP G6-R32HKY-12 lamp holder | $3 |
| Temperature sensor | Plastic casing water-proof DS18B20 temperature sensor | $22 |
| Heat grid relay module | 5V 16 channel relay module | $15 |
| Feeder motor driver | ULN2003 stepper motor driver board | $4 |
| Led strip PSU | 12V 5A AC-DC power supply | $13 |
| Heat lamp PSU | 24V 12A 288W AC-DC power supply | $44 |
| Feeder PSU | 5V 500mA AC-DC power supply | $5 |
| Touchscreen (Optional) | ELO Touch Solutions AccuTouch 1790L 17" LCD Open Frame Touchscreen Display | $581 |
| Thermal camera | FLIR A70 Thermal Core w/95º Lens. Image Streaming Configuration | $7,378 |

**Supplementary Table 2**. Price list for arena components. All components, including the visible light cameras, are relatively low cost. An exception to this is the PC which is necessary to facilitate real-time processing. Another exception is the thermal camera which is not required if thermal monitoring is not a part of the experimental design.
