# Supplementary material for: ReptiLearn: An automated home cage system for behavioral experiments in reptiles without human intervention
Source: PLoS Biol. 2024 Feb 29;22(2):e3002411. doi: 10.1371/journal.pbio.3002411 (PMC10931465; doi:10.1371/journal.pbio.3002411)
Supplement: Attachment — Submitted filename: Response to referees.docx [file pbio.3002411.s013.docx]

**Response to referees and editorial requests**

We thank the referees and the editor for their careful read of the manuscript and their helpful comments and constructive suggestions. We attempted to answer each and every one of their comments and revise the manuscript accordingly. For clarity, we colored the referee’s comments in blue, our responses in black, and the changes we made in the manuscript in red. We also numbered the comments of each reviewer for reference. We believe these revisions considerably improved the manuscript.

**Reviewer 1**

We thank the reviewer the support and we are glad the reviewer found our manuscript “very well written”, “well detailed” and our experimental system ”impressive”, “first of its kind” and “a valuable system with lots of promise”

1. “The only major comment I have is to consider the structure of the methods and results. It's tricky given the nature of this study, but I found the results to be a real mixture of methods and results. I suggest adding subheadings to make the paper easier to follow and restructuring methods and results in a more traditional format.”

To make the manuscript easier to read and more traditionally structured we’ve made the following changes: 1) We added subheadings to the results section. 2) We move technical information from the results to the Methods section and restructured the results to make them more compact. 3) We restructured the order of Methods sections correspondingly and added subtitles to make the different sections more easily searchable.

2. I have the following relatively minor suggestions.

Abstract, P9. "In contrast, natural behaviors evolve over multiple time scales and under minimally constrained conditions in which actions are selected through bi-directional interactions with the environment and without human intervention." I see what you are saying but the natural world comes with a whole other set of constraints (threat of predation, varying food availability, etc). I would not use "minimally constrained conditions".”

To make our statement more accurate we removed the term “minimally constrained”. The revised sentence now reads: “In contrast, natural behaviors evolve over multiple time scales during which actions are selected through bi-directional interactions with the environment and without human intervention.”

3. “When you say "more natural experimental designs" do you mean more representative of what happens in nature?”

Indeed. We rephrased the sentence to make it clearer. It now reads: “Recent technological advancements have opened up new possibilities for experimental designs that more closely mirror natural behaviors by replacing stringent experimental control with accurate multidimensional behavioral analysis.”

4. “Introduction. P 10 survival instead of survivability”

Corrected.

5. “I'm not sure I would say that behavioural ecology is aimed at capturing the full range of behaviours in the wild. It's more about studying behaviour in an evolutionary/fitness framework. Ethology is more likely to capture the full range of behaviours in the wild.”

We removed the term “behavioral ecology”. This term does not appear in the revised introduction since we replaced the focus on differences between research fields with a focus on challenges across fields (see comment 1 of reviewer 2).

6. You mean bi-directional interactions quite frequently. It might be worth clearly explaining what you mean by this and offering some examples at the start.

To increase clarity, we defined bi-directional interactions at beginning of the introduction and moved our example of bi-directional interactions from the results to the introduction. This sentence added to the introduction now reads:

“In such bi-directional interactions, animals constantly receive information from the environment (e.g. perceived prey) and act upon it (e.g. attack the prey), but at the same time, their actions change the environment (e.g. their prey escapes due to their approach (7))”

7. “1980s not 1980's”

Corrected.

8. "…models such as species from the reptilian and amphibian classes." I would just say …models such as amphibians and reptiles.

Corrected.

9. "Correspondingly, the cognitive capacities of reptiles and amphibians remain poorly understood and research linking behavior with neurophysiology is scarce (39).." You could also cite Szabo et al review of reptile cognition in Biological Reviews.

Review added.

10. In the intro you could reference some of the automated cognition work going on in the wild, such as Lucy Aplin's work on tits.

Reference to this work was added.

11. Results. Pg 1. The first sentence has no relationship to the sentences that follow. Need some restructuring.

To increase coherence and flow, the first sentence (which was indeed out of place) was removed and integrated into the last paragraph of the introduction.

12. How about subheadings in the Results? It would make the paper easier to follow and give it a more defined structure.

To improve clarity, we’ve added subheadings to the results section.

13. “Larvae not larva”

Corrected.

14. “Pg 12 Results. Postures can also be related to temperature, they can be influenced by environment.”

We integrated this information into the text which now reads: “Locomotion and posture changes provide valuable information about the animal’s behavioral states and strategies (51) and are linked to environmental factors such as temperature (52).”

15. P 14, pg 1 have a look at Devi Stuart-Fox's publications on Pogonoa temperature using thermal cameras. I'm also not clearly understanding how you verified body temperature? A common method is to measure cloacal temperature and skin temperature at the same time and use regression to predict body temperature from skin temperature.

We’ve added a reference to smith et al (2016) and expanded on the conversion between skin temperature and core temperature across species and specifically in P. Vitticeps.

“These data reflect skin temperatures but can be converted to core temperature using a simple calibration, as previously performed for Pogona Vitticeps (66) (Figure S2C) and additional lizards of similar size (67,65,68).”

As a proof of concept, we also added a supplementary figure (Figure S2C – see below) in which we performed a conversion to core temperatures based on smith et al (2016), as suggested by the reviewer:

“Further, the monotonic relation between skin and core temperature (with approximately linear relation at temperatures of up to ~35°C) allows estimating core temperatures from skin temperatures (67,65,66,68) (Figure S2C).”

This information was added to methods: “To illustrate the conversion from skin temperature (ST) to core temperature (CT), we used the calibration in (92). Specifically, core temperature was estimated (Figure S2C) using a second order polynomial fit (CT(T) = p1* ST 2 + p2* ST + p3, p1=0.0159; p2=1.7066, p3=-6.1034).”

We also emphasized in the relevant parts of the results and methods sections that we measured skin temperature and not core temperature.

16. "ReptiLearn also includes hardware and software for interacting with reptiles visually and via touch (58)." Be specific by what you mean when referring to interaction via touch.

We rephrased this section and added an example to increase clarity:

“For presenting images or videos, we integrated into ReptiLearn a web application that can be used to display and animate custom stimuli on any number of touch screens (Figure 1B). Animal screen touches can be registered and relayed back to the system for real-time feedback to the displayed stimuli or to other triggerable arena components. This module can be used, for example, for displaying prey items on the screen and for logging lizard screen strikes.”

17. P 15 you say 2 s and then 5 seconds—be consistent.

The two values correspond to different parameters. To clarify this, we rephrased the caption of figure 4: “Following the lizard’s detection in the rewarded location (entering the rewarded location and staying there for 2 seconds), a LED was turned on (blinks for 5 seconds), after which a live prey was delivered.” and in the results: “Specifically, upon entering the reward location and staying there for 2s (wait period), the LED in the rewarded location started to blink and 5 seconds later it was turned off, and a command was sent to dispense the reward”

18. The results section has a lot of methods mixed in. For example, p 15 pg 2. You may need to carefully consider the methods and results and rework and restructure them to be more complementary.

We restructured the methods and results. See response to comment #1.

19. Instead of saying Pogona you should say P. vitticeps.

Corrected.

20. P 16, nonsignificant, not "insignificant".

Corrected.

21. Fig. 1 d. Hopefully this will be bigger in publication or that we can open into a bigger format. As presented, it's too small to see much or read any of the text.

We apologize for the quality. We will make sure that the published version will be in high resolution with visible details.

22. Fig. 4. Is the yellow area actually demarcated in the arena or is it spatially mapped on the camera system?

The yellow area (or any rewarded area) in not marked physically on the area. It is only mapped on the camera (defined in the image analysis algorithm). We clarified this in the Results: “Rewarded locations were only defined in the video analysis algorithm and were not physically marked in the arena.“ and in the caption of Figure 4: “The lizard was conditioned to visit a predefined area in the arena (yellow circle, not physically marked in the arena and invisible to the lizard).”

23. Discussion. Section on temperature, p 21-22. In reptile studies, it's also very important to know an animal's preferred body temperature because this is crucial for designing behavioural and cognitive studies and for understanding its natural history. We typically do this in a thermal gradient from very hold to very hot and measure the animals temperature using a probe/data logger, etc. You could tout your system more directly as an alternative method of measuring Tpref. You kind of already say this but not explicitly. (There is a quite vast literature on lizard thermal biology.) In thermal gradients they don't surgically implant sensors but they do insert probes into the cloaca.

We’ve incorporating the reviewer’s suggestion to use the system for estimating the preferred temperature in the discussion: “Using such estimation, our approach could be used for measuring the preferred temperature of freely behaving animal without the need to construct specialized setups (74) by simply averaging over the animal’s temperatures (Figure 3D)”

24. Are there other cognitive tests that you think ReptiLearn could be used for?

We elaborated on potential uses for reptiLearn in the discussion:

“In this study, we demonstrated how ReptiLearn enables systematic investigation of spatial cognition and short-range navigation. This approach can be easily extended to explore additional cognitive capacities. For example, by presenting different auditory or visual stimuli on the screen and differentially linking them to rewards, we can gain insight into the sensory processing capacities of reptiles (86). Temperature manipulation and monitoring during these tasks will allow linking cognitive performance with thermal regulation. Long-term performance monitoring can provide insight into memory prioritization and long-term storage capacities…”

25. Bottom of p 22 and maybe other places. It's better not to use "cold-blooded" because many ectotherms, such as lizards, have high preferred body temperatures, sometimes in excess of 40 C.

We’ve changed “cold blooded” to “ectotherms” throughout the manuscript.

26. P 22, bottom "Despite pioneering work in the field.." I would cite Szabo et al review of reptile cognition in Biological Reviews.

Reference added.

27. Methods section

Spatial learning task. Can you provide details of the actual task? The majority of the details are in Figure 4 in Results and many of the details are also scattered in results.

In the revised manuscript, we collected all the information about the Spatial learning task and placed it in method. This section now reads:

“**General task conditions.** Animals were moved from the animal house to the arena near the end of the light period and remained there for the duration of the experiment (two to three weeks). The arena contained a shelter, a water dish, and a basking area. Before starting the experiment, each animal was cleaned inside an open box filled with shallow water to prevent excessive dirt from reducing position-tracking accuracy. Subsequently, animals were placed in the dark arena, simulating nighttime, and the experiment protocol began the following day (at 7 a.m.). During all experiments, lizards received a vegetable meal every seven days. They were given live food manually in case they did not reach a minimum of 10 worms every three days. The experiment was fully automated by the experiment python module (see system/experiments/loclearn2.py in the ReptiLearn repository). The LED strip and the heat lamp were automatically turned on from 7 a.m. to 7 p.m. daily.

**Task structure.** The task consisted of three blocks. In the first block, lizards were rewarded for entering the feeder area, and in the second and third blocks for entering area 1 and 2, respectively (Figure 4B). Specifically, upon entering the reward location and staying there for 2s (wait period), the LED in the rewarded location started to blink and 5 seconds later it was turned off, and a command was sent to dispense the reward. In pilot tests, we found that lizards positioned themselves on the edge of rewarded areas such that slight movements resulted in repeated crossing into the rewarded areas. To solve this, we required animals to exit the rewarded area (and wait 20 seconds starting from LED blink onset) before entering again to receive another reward. Rewarded locations were only defined in the video analysis algorithm and were not physically marked in the arena. The transition between blocks was automatic after 60 rewards were received, or after six days, whichever occurred first.”

28. You say it is low cost. I understand that costing will vary based on types of cameras, etc., but it would be good to have a ballpark of what that would be.

To provide an estimate of the cost, we’ve added a supplementary item (Table S2) with the prices of the components.

**Reviewer 2**

We thank the reviewer the support and we are glad that the reviewer found our approach “exciting”, “timely and innovative”.

1. “The introduction presents a rather old fashioned view of how different fields approaches behaviour and cognition. I believe most of these fields see the benefit from the other, often collaborate and the introduction should be set up to frame the key challenges rather than different approaches - all approaches would benefit from this methodological advancement.”

We agree with the reviewer that both fields are mutually beneficial and that both could gain from new experimental approaches. To address this comment, we rewrote the first two paragraphs of the introduction. Instead of focusing on contrasting the approaches in ethology and psychology, in the revised manuscript we introduce the key challenges in studying behavior across disciplines and the common need for new experimental platforms.

2. “I would like to see the data from the spatial task and reversal presented in a traditional way so researchers can see clearly how the data compares to standard data, particularly in relation to learning rates. Consideration of how long this sort of data might take to collect using a standard paradigm would also be extremely useful.”

To show our results in a more traditional way, we added a supplementary plot (Figure S4 in the revised manuscript). Since in our approach, animals behave freely, there are no defined trials which we can score. We therefore quantified the changes in behavior (spatial correlations in entry rate - Figure 4G - see below) over days which provides information about the learning rate.

In addition, in the revised manuscript we discuss the point raised by the reviewer about the difference between our approach and more traditional approaches:

“In contrast, our paradigm allows unrestricted animal movement throughout the entire experiment eliminating possible biases introduced by human handling. Further, animals choose when to engage in the task as part of their uninterrupted behavioral routines. The lack of defined trials prevents calculating trial statistics but provides information about animal activity preferences (Figure 4F). This approach results in high engagement over consecutive days during which animals successfully achieved the task tens of times per day (Figure S3). While this rate is hard to compare to paradigms with defined trials, the number of reward events is comparable and in many cases higher than in conventional trial based experiments (82,83,78).”

**Reviewer 3**

We thank the reviewer for the support, and we are glad that the reviewer thinks our manuscript “presents a significant contribution to the field of reptile behavior studies”, finds our approach

“innovative”, our data “compelling” and our manuscript “well-written”.

1. “Introduction

The introduction effectively sets the stage for the need for ReptiLearn. The historical context and the limitations of existing methodologies are well articulated. However, the authors could further sharpen this section by briefly mentioning earlier attempts (if any) to automate reptile behavior studies and how ReptiLearn improves upon these.”

As far as we know, there is no literature describing complete automated solutions for reptiles or amphibians. We now emphasize this in the text in addition to referencing the valuable achievements towards this goal:

“While progress has been achieved in automating specific elements within behavioral tasks for ectothermic vertebrates, or in adapting paradigms designed for other vertebrates (41–43), no complete solution exists for automated behavioral experiments in any reptile or amphibian.

2. “The Methods section is quite detailed, which is commendable. However, it might benefit from additional subheadings or bullet points for better readability, especially in outlining the technical specifications of ReptiLearn.”

To improve the readability of the methods, we divided methods to smaller subsections and added subheadings to all subsections. In addition, we reorganized the paragraphs in a more consistent manner.

3. “Technical specifications

The technical description of ReptiLearn, including the software, hardware, and integration aspects, is thorough. However, it would be helpful to include a discussion on potential technical challenges or limitations one might have encountered and how they were addressed.”

We encountered multiple technical challenges during the construction of the arena and design of the behavioral task. Rather than discussing these challenges collectively in a paragraph, which could be too technical for the reader, in the revised manuscript, we elaborate on difficulties and solutions within the relevant sections of the methods. A few examples are provided here:

“Commercially available aquarium dispensers are suitable for providing live food. However, they are slow and do not provide means for external control. To control the food dispenser and reduce its latency, we modified its control circuit. We connected…”

“…To prevent IR light emitted by heat lamps from flooding images, we covered the Firefly camera lens with a IR cutoff filter attached using custom 3D-printed holders”

“Lamp heating can vary between different manufacturers resulting in different ground temperatures. To control heating intensity, we modified lamp height by attaching 20 cm steel M6 spacer to the steel bars.”

“In some experiments, a larger quantity of worms was needed between refills. This was solved by vertically and horizontally stacking the feeders (up to 30 worms in two vertically stacked feeders and 150 for five horizontally stacked feeders). We vertically aligned stacked feeders…”

“To keep animals motivated, smaller worms are more suitable. We therefore used Tenebrio molitor larvae as rewards. However, these worms may turn to pupa in experiments with low reward rate and long durations of a few days. In such cases, using young Zophobas atratus can extend larval stages (93)”

“In pilot tests, we found that lizards positioned themselves on the edge of rewarded areas such that slight movements resulted in repeated crossing into the rewarded areas. To solve this, we required animals to exit the rewarded area (and wait 20 seconds starting from LED blink onset) before entering again to receive another reward.”

“However, these worms may turn to pupa in experiments with low reward rate and long durations of a few days. In such cases, using young Zophobas atratus can extend larval stages (93)”

4. “In the results section, the data on Pogona vitticeps is compelling. However, the paper would benefit from additional discussion on the generalizability of these results to other reptile species, given the diversity within this class.”

We added a discussion about generalizability across reptiles:

“We demonstrated the usefulness of ReptiLearn in one species out of the large and diverse class of reptiles. While our approach will not fit all reptiles, many of the challenges we tackled are not unique to *P. Vitticeps*. The strong dependence on temperature, weak dependence of food and water reward, preference for eating live prey, low movement rates and engagement in repeated trials, and slow habituation to human handling are shared across many ectothermic vertebrates (60,44). Thus, studies in a wide range of species can potentially benefit from our approach.”

5. “The integration of temperature control in your behavioral experiments is innovative. However, more detail on how these temperature conditions were optimized for different reptile species (if tested) would be beneficial.”

We did not optimize temperature conditions for other reptile species, but our arena could be potentially used for testing species-specific preferences. We discuss this in the revised manuscript:

“Using such estimation, our approach could be used for measuring the preferred temperature of freely behaving animal without the need to construct specialized setups (74) by simply averaging over the animal’s temperatures (Figure 3D).”

6. “Can the authors comment on the reward delivery latency? Is this delay appropriate for most rewarded learning tasks?”

We added information about the relevance of the feeder’s reward latency and its possible bridging by additional cues:

“To integrate automatic delivery of live food (e.g., Tenebrio molitor larvae), we modified inexpensive aquarium fish feeders (Methods) to achieved a narrow distribution of reward delays with mean latency of 3.21s (SD=0.54s) (Figure 2F) instead of an original delay of more than 10s. While different tasks may require different latencies, this latency is comparable to manual delivery of rewards in reptiles (55). However, if shorter delay times are needed, visual stimulation devices (single LED illumination, arena LED strip illumination, or stimulation on a touch screen) are implemented in ReptiLearn and can be used for bridging the gap between behavior and reward delivery (as utilized in our spatial task, Figure 4A).”

7. “Statistical analyses

The analysis seems robust, but more information on the statistical methods used for data analysis, particularly in the animal head position tracking and travel distance measurements, would be useful. This would include any assumptions made in your statistical models.”

We added more information to the analysis section. This information included further elaboration on the travel distance measurements and head position tracking:

“**Travel distance measurements.** We measured the travel distance of the animal by cumulatively summing the distances between head position coordinates along pairs of consecutive video frames. To bridge frames with poor YOLOv4 annotation (see above), we linearly interpolated the position in these frames using values in neighboring frames. Lizards can spend long periods of time in the same location. During these times small random fluctuations in YOLOv4 position estimation are accumulated resulting in increased travel distance. To remove such contributions, we used Gaussian filtering on the position coordinates (window size = 51; SD = 17). The filter window size was chosen empirically by plotting travel distance as a function of window size for a range of values during periods of limited movement: Travel distance was observed to drop quickly for small window sizes and nearly saturate for windows larger than 51. To transform the measurements from pixels to centimeters, we multiplied the values by a constant factor, which was calculated by computing the distance between several pixel pairs with known physical distances and averaging the resulting multiplication factors.”

“**Animal head position tracking.** To track the lizards head and determine if it is inside the reinforced area, we trained YOLOv4 (53) - a light-weight convolutional neural network. The model was used to detect the animal’s head bounding box in each top-view camera frame (Figure 2A). Training was performed using a custom dataset consisting of ~2000 grayscale images of P. vitticeps lizards from various angles, cameras, and different backgrounds, as well as ~1400 grayscale images that did not contain a lizard, which were added to reduce the number of false-positive detections. Approximately 800 of these negative examples were images of birds and insects from the Imagenet dataset (90), and the rest were images taken from several empty arenas. The resulting model was able to detect the lizard head in a wide range of images with various individual lizards, camera angles, and arenas (Figure 2D). Each top-view camera frame was resized to 416 x 416 pixels and then processed with the trained model. The output of the model provides a confidence score for each bounding box. Non-max suppression (NMS) with a 0.6 threshold was used to remove overlapping detected bounding boxes with lower scores (53). Additionally, detections with scores lower than 0.8 were discarded. When the output of the NMS algorithm resulted in multiple bounding boxes, we used the box closest to the previous detected one and ignored the others. We used the bounding box's center point to estimate the animal's head position in the arena.”

8. “In the Results and Discussion sections, while they have shown that ReptiLearn is effective for Pogona vitticeps, a more detailed discussion on its effectiveness for studying a broader range of behaviors in reptiles would be beneficial, especially considering the variability in behavioral patterns across different reptilian species.”

Please see response to point #4

9. “Broader implications/future directions

The Discussion section adeptly covers the implications of findings and the potential of ReptiLearn. Expanding on how this system could pave the way for new research areas in reptile behavioral studies would enrich this section.

In the revised version, we expanded on the use of ReptiLearn in other behavioral studies:

“…In this study, we demonstrated how ReptiLearn enables systematic investigation of spatial cognition and short-range navigation. This approach can be easily extended to explore additional cognitive capacities. For example, by presenting different auditory or visual stimuli on the screen and differentially linking them to rewards, we can gain insight into the sensory processing capacities of reptiles (86). Temperature manipulation and monitoring during these tasks will allow linking cognitive performance with thermal regulation. Long-term performance monitoring can provide insight into memory prioritization and long-term storage capacities.”

10. The potential for ReptiLearn's application in evolutionary biology and comparative neurology is exciting. A more detailed exploration of these possibilities, perhaps with hypothetical scenarios or proposed studies, would be valuable.”

In the revised version, we expanded on recent measurements of population dynamics from behaving reptiles the potential contribution of ReptiLearn to these efforts:

“Finally, ReptiLearn facilitates the integration of behavioral assays with neurophysiology to elucidate the brain regions and neuronal population patterns underpinning these cognitive skills (75). Recent neuronal recordings from awake reptiles are beginning to reveal the neuronal dynamics underlying brain states (87,88,75) and visual processing (89). Placing such studies within a behavioral context can shed light on the evolution of cognition and provide a comparative perspective critical for generalized understanding (84,34,35,30,31,36,33).”

11. “Figures:

Your figures are informative and well-integrated into the text.”

Thank you.

12. “For Figure 3, the gray area (corresponding to basking periods) does not appear to show up correctly in my version.”

To increase visibility, we darkened the gray area in Figure 3D.

13. “In summary, your manuscript presents a significant contribution to the field of reptile behavior studies. The development of ReptiLearn is an innovative step forward, providing a new tool for researchers in this field. With some additional details and clarifications, particularly in the Methods and Results sections, your paper could have an even greater impact.”

We thank the review again for the kind words. We believe that the changes we made following the reviewer’s suggestions considerably improved our manuscript.

**Editor comments**

“In light of the reviews, which you will find at the end of this email, we are pleased to offer you the opportunity to address the comments from the reviewers in a revision that we anticipate should not take you very long. This includes **restructuring the organization of the manuscript, reframing the Introduction section and providing additional reporting details for the statistical analyses**. After discussions with the Academic Editor, we also ask that the **revision provides a platform to ease the replication of the set-up to increase its accessibility/user-friendliness**, since the system may be difficult for a non-engineer to implement.”

We attempted to address every point raised by the reviewers and have invested considerable efforts in restructuring the manuscript and providing addition details according to the reviewer guidelines (see above). The additional changes we made following the requests of the editors are detailed below. In addition, we reformatted the panel letter in the figures in accordance with PloS guidelines. Finally, we note we placed emphasis on user friendly explanations in our github repository to ease the system’s replication.

We will then assess your revised manuscript and your response to the reviewers' comments with our Academic Editor aiming to avoid further rounds of peer-review, although might need to consult with the reviewers, depending on the nature of the revisions.”

We are looking forward to the editor’s response.

“*IMPORTANT*

In addition, we would be grateful if you could please provide address the following editorial and data-related requests that I have provided below (A-E):

(A) We would like to suggest the following modification to the title:

“ReptiLearn: an automated home cage system for behavioral experiments in reptiles without human intervention””

The title was changed.

“(B) You may be aware of the PLOS Data Policy, which requires that all data be made available without restriction: http://journals.plos.org/plosbiology/s/data-availability. For more information, please also see this editorial: http://dx.doi.org/10.1371/journal.pbio.1001797.

Note that we do not require all raw data. Rather, we ask that all individual quantitative observations that underlie the data summarized in the figures and results of your paper be made available in one of the following forms:

-Supplementary files (e.g., excel). Please ensure that all data files are uploaded as 'Supporting Information' and are invariably referred to (in the manuscript, figure legends, and the Description field when uploading your files) using the following format verbatim: S1 Data, S2 Data, etc. Multiple panels of a single or even several figures can be included as multiple sheets in one excel file that is saved using exactly the following convention: S1_Data.xlsx (using an underscore).”

-Deposition in a publicly available repository. Please also provide the accession code or a reviewer link so that we may view your data before publication.

Regardless of the method selected, please ensure that you provide the individual numerical values that underlie the summary data displayed in the following figure panels as they are essential for readers to assess your analysis and to reproduce it:

Figure 2B-C, 2E-F, 3B, 3D, 4G-I, S1B, S2

NOTE: the numerical data provided should include all replicates AND the way in which the plotted mean and errors were derived (it should not present only the mean/average values).”

In the revised manuscript we added a supplementary file “S1_Data.xlsx” that includes all individual quantitative observations that underlie the data summarized in the figures. This file includes multiple sheets corresponding to each of the figures and their panels.

We also added a reference to this supplementary file (S1 data) in the data availability statement.

“(C) Thank you for already depositing the source code in Github (https://github.com/EvolutionaryNeuralCodingLab/reptiLearn). We ask that you please link the code deposition to the Zenodo repository (https://zenodo.org/) to ensure that the deposition is given a DOI and long-term maintenance.“

We linked the code to a Zenodo repository (DOI: 10.5281/zenodo.10546737) and added the DOI to the manuscript.

“(D) Please also ensure that each of the relevant figure legends in your manuscript include information on *WHERE THE UNDERLYING DATA CAN BE FOUND*, and ensure your supplemental data file/s has a legend.”

We added a legend to data S1 and a reference to this data in each of the figures.

“(E) Please ensure that your Data Statement in the submission system accurately describes where your data can be found and is in final format, as it will be published as written there.”

We updated the data statement. In now reads:

“All data directly relevant are within the paper or in S1 Data. The ReptiLearn software and code are available from https://github.com/EvolutionaryNeuralCodingLab/reptiLearn (DOI: 10.5281/zenodo.10546737).”
